# Comparison of Antibody Responses to Human Papillomavirus Vaccination as Measured by Three Assays

**DOI:** 10.3389/fonc.2013.00328

**Published:** 2014-01-13

**Authors:** Hilary A. Robbins, Troy J. Kemp, Carolina Porras, Ana Cecilia Rodriguez, Mark Schiffman, Sholom Wacholder, Paula Gonzalez, John Schiller, Douglas Lowy, Sylviane Poncelet, Mark Esser, Katie Matys, Allan Hildesheim, Ligia A. Pinto, Rolando Herrero, Mahboobeh Safaeian

**Affiliations:** ^1^Division of Cancer Epidemiology and Genetics, National Cancer Institute, NIH, Rockville, MD, USA; ^2^HPV Immunology Laboratory, SAIC-Frederick Inc., Frederick National Laboratory for Cancer Research, Frederick, MD, USA; ^3^Proyecto Epidemiológico Guanacaste, Fundación INCIENSA, Guanacaste, Costa Rica; ^4^International Agency for Research on Cancer, Lyon, France; ^5^Center for Cancer Research, National Cancer Institute, NIH, Bethesda, MD, USA; ^6^GlaxoSmithKline Biologicals, Rixensart, Belgium; ^7^MedImmune, Gaithersburg, MD, USA; ^8^PPD Vaccines and Biologics Center of Excellence, Wayne, PA, USA

**Keywords:** human papillomavirus, HPV serology, HPV vaccines, cLIA, SEAP-NA, ELISA

## Abstract

**Background:** Different assays, including the competitive Luminex immunoassay (cLIA), secreted alkaline phosphatase neutralization assay (SEAP-NA), and virus-like particle-based ELISA, are commonly used to measure antibody responses after human papillomavirus (HPV) vaccination. Direct assay comparisons aid interpretation of immunogenicity data evaluated by different assays.

**Methods:** We compared cLIA to SEAP-NA and ELISA among 51 HPV16/18-vaccinated women enrolled in the Costa Rica Vaccine Trial. We tested replicate serum samples collected at months 0, 1, and 12 by HPV16/18 cLIA, SEAP-NA, and ELISA. For a subset (*N* = 10), we further tested month 6, 24 and 36 samples. We calculated seroprevalence estimates and Spearman rank correlation coefficients comparing cLIA to SEAP-NA and ELISA.

**Results:** After one vaccine dose, seroprevalence by SEAP-NA and ELISA was 100% (both HPV16 and HPV18), and by cLIA was 96% (95% CI 87–100%) for HPV16 and 71% (95% CI 56–83%) for HPV18. Seroprevalence was 100% by all assays after three doses. Correlation between assays was high after one vaccine dose [cLIA/SEAP-NA ρ = 0.91 (HPV16) and ρ = 0.86 (HPV18); cLIA/ELISA ρ = 0.84 (HPV16) and ρ = 0.74 (HPV18); all *p* < 0.001] and remained high through month 36. Ratios of mean antibody levels to seropositivity cutoffs at month 36 were lower for cLIA than for SEAP-NA or ELISA, particularly for HPV18 (HPV18 ratio for cLIA 1.9, SEAP-NA 3.5, ELISA 3.4).

**Conclusion:** Though correlation between cLIA and SEAP-NA/ELISA is high and stable after vaccination, the assays differ in scale and sensitivity, with notable differences after one vaccine dose and for HPV18. Our results demonstrate that comparisons of antibody responses to HPV vaccination measured by different assays are approximate, and must consider biological and technical differences between assays.

## Introduction

Two human papillomavirus (HPV) virus-like particle (VLP) vaccines, the bivalent (HPV16/18) Cervarix^®^ and quadrivalent (HPV6/11/16/18) Gardasil^®^, are licensed for prevention of cervical cancer and related lesions (1). Four-year efficacy of both vaccines approaches 100% among HPV-naïve women for prevention of high-grade lesions related to HPV types 16 and 18 (2, 3), which together cause 70% of cervical cancers (4).

Neutralizing antibody responses are believed to be the primary mechanism of vaccine-induced protection (5), but different type-specific assays are used to measure them. For Cervarix^®^, the principal assay for measuring immunogenicity has been the VLP-based ELISA, which measures neutralizing and non-neutralizing antibodies of one immunoglobulin class (typically IgG). Neutralizing responses to Cervarix^®^ have been measured using the secreted alkaline phosphatase neutralization assay (SEAP-NA), which broadly and directly measures neutralization potential (6). For Gardasil^®^, the proprietary competitive Luminex immunoassay (cLIA) is primarily used, which measures neutralizing antibodies that compete for binding to one VLP epitope (7).

Measured antibody responses are important, as they are used as evidence of vaccine immunogenicity, but results vary by assay. When different vaccines are evaluated using different assays, variability in results may be due to assay differences or due to true differences in immunogenicity (8). To facilitate interpretation of measurements by different assays, direct comparisons of assays have been published in both natural infection and vaccination contexts (9–12). A previous report by our group compared SEAP-NA to ELISA after vaccination with Cervarix^®^, finding high correlation between the two assays (10). Here, we extend these results by presenting a detailed and longitudinal post-vaccination comparison of cLIA to SEAP-NA and ELISA.

## Materials and Methods

We evaluated women selected from the HPV vaccine arm of the Costa Rica Vaccine Trial (CVT) (13), in which participants were vaccinated with Cervarix^®^ at months 0, 1, and 6. For the present study, we combined two groups of women sampled for previous serological studies where HPV16/18 SEAP-NA and ELISA testing were already performed (10, 14, 15). The first group included 50 randomly sampled women, and the second group included 12 women sampled with the requirement of no cervical infection with HPV types 16, 18, 31, 45, or 58 at baseline (month 0). We further tested replicate sera from these 62 women collected at months 0, 1, and 12 by HPV16/18 cLIA; for the smaller group, we additionally tested samples from months 6, 24 and 36. We excluded four women who received fewer than three vaccine doses and five for whom any of the assays failed, and further excluded two women with anomalous results in post-vaccination samples. One woman was seronegative by all assays at month 12 despite a normal antibody response at other time points; we suspect this may reflect a sample retrieval error. The second woman never seroconverted by HPV16/18 cLIA after vaccination; this may indicate a technical error but does not imply a vaccine failure as other assays showed the expected antibody response. As results did not differ by group, we conducted analyses among a combined analytical sample of 51 women, equal to 62 less 11 exclusions.

The SEAP-NA was performed at the HPV Immunology Laboratory, SAIC-Frederick Inc., as described (6, 10), with results reflecting the mean of at least duplicate testing (maximum of 15 runs) for each sample. Neutralization titers were calculated by linear interpolation and were defined as the reciprocal of the dilution that reduced SEAP activity by 50% compared to control wells. We used the laboratory-determined seropositivity cutoff of 10, which reflects our lowest dilution. The VLP-based ELISA was performed at GSK Biologicals as described (16), with results reflecting the mean of between 1 and 5 runs per sample, and laboratory-determined seropositivity cutoffs of 8 EU/mL (ELISA units per milliliter) (HPV16) and 7 EU/mL (HPV18) were used. Data describing our ELISA (10) and SEAP-NA (10, 14, 15) results have been published. The cLIA was performed at PPD Vaccines and Biologicals as described (7), with use of standard curves to convert mean fluorescence intensities to arbitrary milli-Merck units per milliliter (mMU/mL). Laboratory-determined cutoffs of 20 mMU/mL (HPV16) and 24 mMU/mL (HPV18) are based on an algorithm maximizing the distinction between “likely negative” and “likely positive” samples (17).

We performed statistical analyses separately for HPV16 and HPV18. Among the combined sample of 51 women, at months 0, 1, and 12, we compared cLIA to SEAP-NA and ELISA using seroprevalence estimates, scatter plots, and Spearman rank correlation coefficients. Among the subgroup of 10 women (12 less 2 exclusions), where we additionally had assay data at months 6, 24 and 36, we plotted individual antibody levels by each assay over time. To quantify inter-assay correlation beyond month 12 (i.e., beyond time points available in the larger sample), we also calculated Spearman coefficients at month 36 among these 10 women. For graphical presentation and calculation of correlation coefficients, values below assay lower limits of detection (LLODs) were assigned a value of 1/2 (LLOD).

## Results

**Figure 1:**
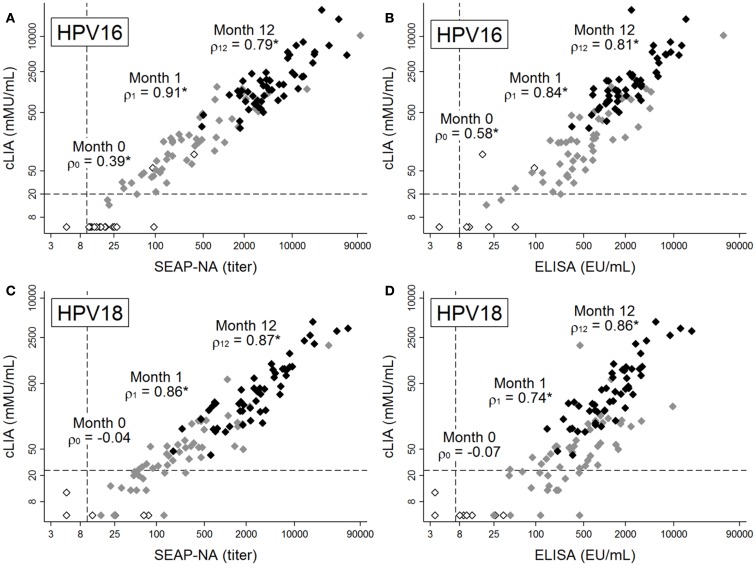
**Antibody levels for HPV16 (A,B) and HPV18 (C,D), as measured by cLIA vs. SEAP-NA (A,C) or cLIA vs. ELISA (B,D)**. Levels were measured at months 0 (white markers), 1 (gray markers), and 12 (black markers) among 51 women in the Costa Rica Vaccine Trial who received HPV vaccine doses at months 0, 1, and 6. Spearman rank correlation coefficients between pairs of assays are displayed for each time point, with asterisks denoting statistical significance (all *p* < 0.005). Dashed lines represent laboratory-determined seropositivity cutoffs. Note differing scales for *x*- and *y*-axes, as assays use different scales for measurement.

**Figure 2:**
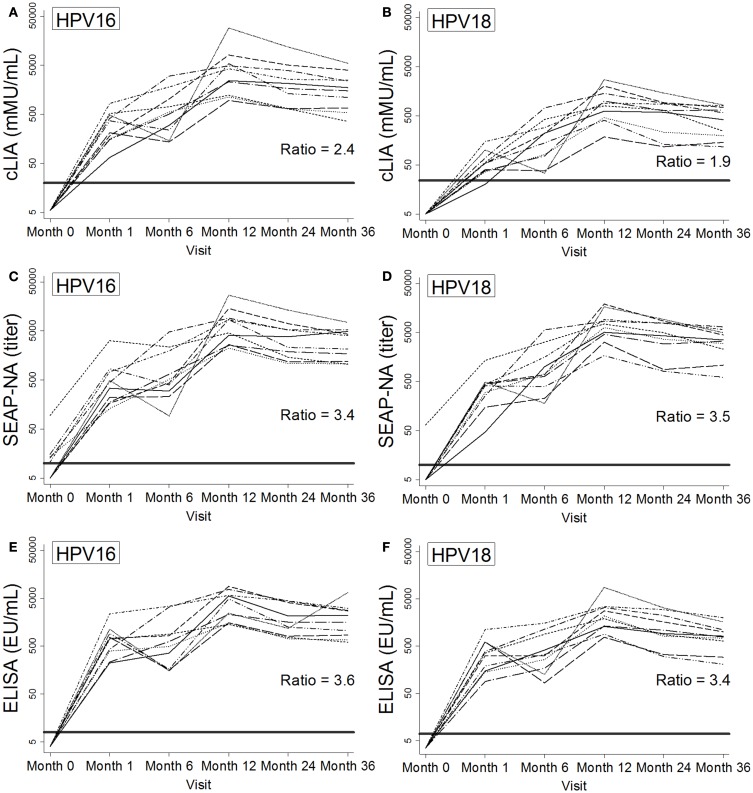
**Individual patterns of antibody levels over time as measured by cLIA (A,B), SEAP-NA (C,D), and ELISA (E,F) among 10 women in the Costa Rica Vaccine Trial who received HPV vaccine doses at months 0, 1, and 6**. Patterns are shown for HPV16 **(A,C,E)** and HPV18 **(B,D,F)**. Bold solid lines represent seropositivity cutoffs, and ratios at 36 months were calculated as the mean of log-antibody levels to the log of the corresponding seropositivity cutoff. HPV DNA negativity for types 16, 18, 31, 45, and 58 at month 0 was required for selection.

HPV16/18 antibody levels increased after vaccination as previously reported by SEAP-NA and ELISA (10, 14, 15) and by cLIA (Figures 1 and 2). At 1 month after the first vaccine dose, all women were HPV16 and HPV18 seropositive by SEAP-NA and ELISA (Table 1). By cLIA, at month 1, 96% (95% CI 87–100%) and 71% (95% CI 56–83%) were seropositive for HPV16 and HPV18, respectively. At month 12, all women were seropositive for both HPV types by all three assays.

**Table 1:** **Seroprevalence by HPV type, assay, and visit among 51 women in the Costa Rica Vaccine Trial**.

HPV type	Assay	Seroprevalence and 95% confidence interval^a^ (%)		
		Month 0	Month 1	Month 12
HPV16	cLIA	4 (0–13)	96 (87–100)	100 (93–100)
	SEAP-NA	33 (21–48)	100 (93–100)	100 (93–100)
	ELISA	12 (4–24)	100 (93–100)	100 (93–100)
HPV18	cLIA	0 (0–7)	71 (56–83)	100 (93–100)
	SEAP-NA	8 (2–19)	100 (93–100)	100 (93–100)
	ELISA	20 (10–33)	100 (93–100)	100 (93–100)

*Seropositivity was defined according to laboratory cutoffs (see Materials and Methods). HPV vaccine doses were administered at months 0, 1, and 6*.

*^a^For estimates where seroprevalence is 0 or 100%, a one-sided 97.5% confidence interval is shown*.

Prior to vaccination, when average antibody levels were very low or below detection limits, correlation between cLIA and SEAP-NA was moderate for HPV16 (ρ = 0.39, *p* = 0.004, Figure 1A) and not present for HPV18 (ρ = −0.04, *p* = 0.774, Figure 1C). Results were similar for cLIA and ELISA (ρ = 0.58, *p* < 0.001 for HPV16; ρ = −0.07, *p* = 0.629 for HPV18, Figures 1B,D). Correlation was high after one vaccine dose (i.e., at month 1), with Spearman coefficients of 0.91 (HPV16) and 0.86 (HPV18) for cLIA and SEAP-NA, and coefficients of 0.84 (HPV16) and 0.74 (HPV18) for cLIA and ELISA (all *p* < 0.001). Correlation remained high 6 months after the third dose (i.e., at month 12), with Spearman coefficients of 0.79 (HPV16) and 0.87 (HPV18) for cLIA and SEAP-NA, and coefficients of 0.81 (HPV16) and 0.86 (HPV18) for cLIA and ELISA (all *p* < 0.001). Discordant samples (i.e., samples seronegative by one assay but seropositive by the other) were present only at months 0 and 1, and were seronegative by cLIA but seropositive by either SEAP-NA or ELISA in all cases (Figure 1). Using data at month 36 (*N* = 10), when plateau antibody levels are expected, correlation coefficients were 0.89 (HPV16, *p* = 0.001) and 0.83 (HPV18, *p* = 0.003) for cLIA and SEAP-NA, and 0.96 (HPV16, *p* < 0.001) and 0.92 (HPV18, *p* < 0.001) for cLIA and ELISA (results not shown).

Antibody kinetics throughout and after vaccination were largely similar across HPV types, assays, and the 10 women studied (Figure 2), with increases in antibody levels throughout vaccination and a gradual decline after month 12. For HPV16/18 SEAP-NA and ELISA, levels were well above seropositivity cutoffs beginning at month 1 (Figures 2C–F). For cLIA, particularly for HPV18, levels were lower and closer to the seropositivity cutoff (Figures 2A,B). To quantify this observation at our final time point (month 36), we calculated the ratio of the mean of log-antibody levels to the corresponding log-cutoff (log-antibody_mean_: log-cutoff). For HPV16, these ratios were 2.4 for cLIA, 3.4 for SEAP-NA, and 3.6 for ELISA. For HPV18, these ratios were 1.9 for cLIA, 3.5 for SEAP-NA, and 3.4 for ELISA.

## Discussion

Different assays are used to monitor antibody responses following HPV vaccination, with ELISA or SEAP-NA typically used for Cervarix^®^ and cLIA for Gardasil^®^. To aid interpretation of immunogenicity data, we directly compared cLIA-measured responses to ELISA- and SEAP-NA-measured responses after vaccination with Cervarix^®^. Correlation between cLIA and both SEAP-NA and ELISA was high beginning after one vaccine dose, with similar antibody kinetics across assays. However, levels relative to seropositivity cutoffs were lower by cLIA than by SEAP-NA or ELISA, particularly for HPV18.

The cLIA, SEAP-NA, and ELISA are technically different assays and measure different aspects of the antibody response (8). The cLIA measures neutralizing antibodies of all immunoglobulin classes that compete for binding to a specific VLP epitope (V5 for HPV16, J4 for HPV18) by evaluating the strength of a fluorescent signal produced by binding monoclonal antibodies (7). SEAP-NA measures a reporter gene product which is expressed when HPV pseudovirions infect susceptible cells, such that decreases in expression reflect overall serum neutralizing potential (6), which is assumed but not required to be antibody-mediated. ELISA measures one class of antibodies (typically IgG) that bind to a fixed VLP antigen by measuring the activity of an enzyme conjugated to a secondary antibody (16). These assays use different scales in terms of measurement, and further differences exist in the structure and production of the VLPs for each assay. The directed nature and more stringent cutoff of cLIA affords it lower sensitivity than SEAP-NA or ELISA (9); further, within the cLIA, the HPV18 J4 epitope may be less immunodominant than the HPV16 V5 epitope.

In our study, the lower sensitivity of cLIA was likely responsible for a number of discordant samples after vaccination. At month 1, approximately 4% of samples were HPV16 seropositive by ELISA and SEAP-NA but HPV16 seronegative by cLIA. The proportion of discordant samples was larger (29%) for HPV18 at the same visit, likely reflecting lower immunodominance of the HPV18 J4 epitope than the HPV16 V5 epitope. Relative to seropositivity cutoffs, HPV18 antibody levels were substantially lower by cLIA than by SEAP-NA or ELISA.

Despite assay differences, we observed high and statistically significant correlation between cLIA and both SEAP-NA and ELISA beginning after one vaccine dose and extending to month 36, with similar antibody kinetics across HPV types and assays. A previous study, comparing cLIA to an in-house SEAP-NA, found correlation coefficients of 0.67 (HPV16) and 0.91 (HPV18) 1 month after the third dose of Gardasil^®^ (12). Another study conducted among Gardasil^®^ vaccinees compared cLIA to a total IgG Luminex immunoassay, which is biologically similar to but technically different from our ELISA (18). Results were similar to our cLIA/ELISA comparison, with correlation coefficients at months 7, 24, and 48 of 0.66, 0.90, and 0.91 for HPV16, and 0.84, 0.88, and 0.89 for HPV18. Our group previously reported high correlation between SEAP-NA and ELISA for combined 1- and 12-month measurements, with coefficients of 0.91 (HPV16) and 0.85 (HPV18) (10).

In conclusion, though agreement between assays is not high prior to vaccination (9), among vaccinated women measurements by cLIA, SEAP-NA, and ELISA correlate well and have similar patterns over time. However, the assays differ in scale and sensitivity, with notable differences after one vaccine dose and for HPV18. Comparisons of antibody responses to HPV vaccination measured by different assays are therefore approximate, and must consider biological and technical differences between assays.

## Conflict of Interest Statement

One or more of the authors is employed by a commercial company. These include: Sylviane Poncelet, GlaxoSmithKline Biologicals; Mark Esser, MedImmune; Katie Matys, PPD Vaccines and Biologics. John Schiller and Douglas Lowy report that they are named inventors on US Government-owned HPV vaccine patents that are licensed to GlaxoSmithKline and Merck and for which the National Cancer Institute receives licensing fees. They are entitled to limited royalties as specified by federal law. The other authors declare that they have no conflicts of interest.
